# Risk factor patterns and vascular health in children with incident hypertension: The ExAMIN Youth SA study

**DOI:** 10.1038/s41371-025-01074-w

**Published:** 2025-10-02

**Authors:** Jonathan Nsamba, Danielle Swanepoel, Leandi Lammertyn, Wayne Smith, Ruan Kruger

**Affiliations:** 1 Hypertension in Africa Research Team (HART), Faculty of Health Sciences, North-West University, Potchefstroom, South Africa; 2 MRC Extramural Research Unit for Hypertension and Cardiovascular Disease, North-West University, Potchefstroom, South Africa

**Keywords:** Risk factors, Cardiovascular biology

## Abstract

Risk factor exposure during childhood contributes to the early onset of cardiovascular disease and clusters with incident hypertension. We investigated cardiovascular risk factor patterns in preadolescent children, stratified by BP status, and their associations with macro- and microvasculature measures. We included children (n = 1043, ages 5–9 years) from the ExAMIN Youth SA study. Measurements included anthropometry, cardiorespiratory fitness, dietary intake, office BP, central pulse wave velocity (PWV), central retinal artery (CRAE), vein equivalent (CRVE), and their ratio (AVR), to identify factor patterns with exploratory factor analysis. We identified three factor pattern scores (FPS). FPS1 (chips, sweets, fast foods, and cookies/cake) and FPS2 (fruits, meat, milk, and socioeconomic status) were identified in both the normotensive blood pressure (BP) and incident hypertension groups, with the exceptions of fruits being absent in the incident hypertension group and fast foods absent in the normotensive group. FPS3, characterised by BMI, diastolic BP, and systolic BP, was observed only in the normotensive group. PWV associated with FPS3 (β = 0.372, p < 0.001) in the normotensive but with FPS2 (β = 0.197, p = 0.045) in the incident hypertension group. CRAE (β = −0.224, p = 0.001) and AVR (β = −0.26, p < 0.001) inversely associated with FPS3 in normotensive but with FPS1 in the incident hypertension (CRAE: β = −0.343, p < 0.001; AVR: β = −0.274, p < 0.001). CRVE was positively associated with FPS3 (β = 0.194, p = 0.002) in the normotensive group. Exposure to unhealthy dietary patterns in childhood compromises vascular health in the context of incident hypertension, beginning as early as five years of age.

## Introduction

Cardiovascular diseases (CVD) remain the leading causes of morbidity and premature mortality in the world, with hypertension as the primary risk factor for CVD [1]. The prevalence of primary hypertension in children has increased significantly [2]. A recent meta-analysis reported an elevated BP prevalence of 22% among children and adolescents in Africa under 18 years [2]. The early onset of hypertension is associated with CVDs in later life. A study [3] reported the association of childhood risk factors such as elevated BP, obesity, and high cholesterol with fatal and non-fatal cardiac events occurring before the age of 47 years. These findings emphasise the critical need to address modifiable risk factors early in life to prevent the progression of CVD and adverse outcomes in early adult life.

Modifiable risk factors such as obesity, and unhealthy dietary intake collectively contribute to the burden of CVD [4]. These risk factors are already present in childhood [5] and may contribute to the early deterioration of macro- and microvascular health in children [6, 7]. Such vascular changes are associated with elevated blood pressure and may contribute to the onset of premature cardiovascular disease.

Target organ injury markers such as pulse wave velocity (PWV) and retinal vessel calibres (CRAE: central retinal artery equivalent; CRVE: central retinal vein equivalent and their ratio, AVR: arterio-venous ratio) have shown to have predictive value for cardiovascular events in adults [8, 9]. The use of these markers in childhood may improve our understanding of the early mechanisms involved in CVD development at an early age. In children, obesity and lower cardiorespiratory fitness are associated with higher PWV, wider CRVE, smaller CRAE, and lower AVR [10–12], but no study has explored combined risk factor clusters, including BMI and physical activity, in relation to BP status and target organ injury markers. Although the individual impact of risk factors on hypertension is well-known, elevated BP in children is rarely isolated, and the combined effects of risk factor clusters on macro- and microvasculature remain underexplored [11]. This study aims to identify risk factor patterns and their associations with markers of vascular health in South African primary school-aged children stratified by blood pressure status.

## Methods

### Study design and participants

The Exercise, Arterial Modulation, and Nutrition in Youth South Africa (ExAMIN Youth SA) study is a multidisciplinary, observational cohort and analytical study that was developed to better comprehend the complicated mechanisms that are associated with the origin of early cardiovascular modification in children [12]. In this analysis, we included 1043 participants out of the original cohort of 1103 participants who were assessed at baseline [13]. A total of 37 participants were excluded for having missing data, and 23 belonged to other ethnicities. Participants were recruited from public primary schools in the North-West province of South Africa.

### Basic procedures

The detailed study procedures have been published previously in the ExAMIN Youth SA protocol paper [12]. In brief, all eligible children received an information letter to take home to their parents or guardians. Participation was entirely voluntary; neither children nor parents were obligated to take part. Parental permission was sought through a structured consent process after families were given the opportunity to ask questions and receive full information about the study aims and procedures. Only children with completed parental consent and child assent forms were included in the study. Approval was obtained from the schools to conduct the research on their premises.

In addition to physical measurements, parents or guardians completed a questionnaire that captured information on the child’s age, sex, and parental education. The questionnaire also included items on socioeconomic and health-related variables. Socioeconomic status (SES) was quantified using a composite mean score derived from household income, parental education level, and employment status, with higher scores indicating higher SES.

### Anthropometric and cardiorespiratory fitness measurements

All anthropometric measurements in this study were determined in accordance with the International Society for the Advancement of Kinanthropometry [14]. The body weight was taken with a Seca 813 numerical scale to determine weight to the closest 0.1 kg, and height with a Seca 213 stadiometer (Birmingham, United Kingdom) with a perpendicular panel to the closest 1 mm. Sex-adjusted body mass index for age (BMI z-score) was calculated using the World Health Organisation’s AnthroPlus software for children according to the child growth references for children between 5–19 years old [15]. Cardiorespiratory fitness (CRF) was measured by the 20-meter shuttle run test to objectively assess maximal aerobic capacity after a standardised 5-min warm-up [16]. The number of “stages” accomplished, where 1 stage ≈ 1 min, is calculated with a precision of 0.5 stage [14]. The test was done in the morning during school time and not during the winter season. CRF was recorded as the number of shuttles completed.

### Cardiovascular measurements

#### Blood pressure

Office blood pressure and heart rate of all the participants were determined with validated, automated oscillometric paediatric BP monitors [Omron HBP-1100-E, (OMRON Healthcare Co., Ltd. Kyoto, Japan)] [17]. Prior to the BP measurement, the participants were requested to sit relaxed for 3–5 min with their backs supported, right arm horizontally supported, with their feet on the ground, and the cubital fossa at heart level.

Blood pressure was measured using standardised procedures in accordance with the 2017 American Academy of Paediatrics Clinical Practice Guidelines [18] for screening and management of high BP in children and adolescents. Measurements were taken on the right arm using appropriately sized cuffs. BP was measured five times at one-minute intervals, and the mean of the three readings with the least variance was used in analysis [19]. We grouped children according to normal BP range ( < 90th percentile for age, height and sex) and incident hypertension ( ≥ 90th percentile for age, sex and height or >120/80 mmHg).

#### Pulse wave velocity

Validated oscillometric Mobil-O-Graph monitors, along with the HMS Client-Server software package Version 4.7.1 (I.E.M. GmbH, Germany), were used to non-invasively perform pulse wave analysis [20]. These monitors were programmed to complete pulse wave analysis in duplicate using an appropriately sized cuff to the participants’ right arm, while in a sitting position. The aortic PWV was determined through this method [12].

#### Retinal vessels

A Static Retinal Vessel Analyser (SVA-T, Imedos Systems GmbH, Jena, Germany) connected to a fundus camera (Topcon TRC NW8) was used to capture non-invasive and nonmydriatic optic disc-centred retinal fundus images. Each child had two images taken of the right eye at an angle of 45 degrees. Vessel analysis (Visualis 2.80, Imedos Systems GmbH, Jena, Germany) was semi-automatically performed and retinal arteries and veins, running through an extent of 0.5–1 disc diameter from the optic disc margin, were selected. CRAE and CRVE were obtained by averaging the vessel diameters through the Parr-Hubbard formula, and the arterio-venous ratio was then determined (CRAE/CRVE) [21]. The mean of the two images of the right eye was used for CRAE and CRVE. The vessel diameters were expressed in measuring units, where 1 mu equalled 1 μm in the model of Gullstrand’s normal eye.

### Food intake survey

A face-validity food frequency questionnaire was developed and validated [22] to gather dietary data on foods that are generally consumed by South African school children. The food intake survey included four healthy (vegetables, fruits, and fish/meat/poultry/eggs/milk), and six unhealthy food groups (cold drinks, sweets, sugar-sweetened hot drinks, cakes, fast foods, and salty snacks [23], based on the WHO Global School-based Student Health Survey [12].

### Statistical analysis

Statistical analyses were performed with IBM^®^ SPSS^®^ Statistics version 30 software (IBM Corporation; Armonk, New York, USA), and figures were made using GraphPad Prism v5.03. All variables were tested for normality by visual inspection (QQ-plots). Normally distributed data were expressed as mean ± standard deviation, while non-normally distributed variables were expressed as mean sum ranks. Chi-square tests were used to compare the proportions of dichotomous variables. We performed independent t-tests to compare mean values between groups, while the Mann-Whitney U-test was used for food group data.

Factor analysis using the dimension reduction function of SPSS was used to identify risk factor patterns. Principal component analysis was used, and factors with an eigenvalue > 1.5 were retained. The Oblimin rotation method was used to obtain independent factors. A factor loading of ≥0.5 was used to interpret the factor patterns that were automatically calculated by the statistical software and used in further analyses. Pearson and partial correlations (adjusted for age, sex, ethnicity, and PWV was additionally adjusted for heart rate and mean arterial pressure) were performed to determine the associations of PWV, CRAE, CRVE, and AVR with each cardiovascular risk factor pattern in the normotensive and incident hypertension groups.

Multiple regression analysis was performed to test the independent associations of PWV, CRAE, CRVE, and AVR with each cardiovascular risk factor pattern in the normotensive and incident hypertension groups. Covariates considered for entry in the models included age, sex, and ethnicity, and additionally, mean arterial pressure and heart rate with PWV as the dependent variable.

## Results

### Characteristics of the study groups

Table 1 displays the characteristics of the study group stratified by BP ranges. Sex, age and ethnic distribution were similar between the groups (all p > 0.05). The children with incident hypertension had higher anthropometric measures, BP, and heart rate (all p < 0.001) than those in the normotensive group. Children with incident hypertension had higher mean PWV than the normotensive group (p < 0.001). CRAE was narrower and AVR lower in the incident hypertension group (both p < 0.001), whereas no differences were observed for CRVE and dietary intake between the groups.

**Table 1:** Characteristics of the study population stratified by BP categories.

	Normotensive n = 662	Incident Hypertension n = 381	p-value
*Demographics*			
Sex, girls, n (%)	367 (64.7)	200 (35.3)	0.358
Age, years^a^	7.40. ± 0.95	7.50 ± 0.86	0.070
Black ethnicity, n (%)	367 (63.3)	213 (36.7)	0.884
Socioeconomic status score	5.68 (2.65)	5.90 (2.60)	0.271
*Anthropometric measures*			
Body height, cm^a^	122.41 ± 7.78	124.01 ± 7.91	0.002
Body weight, kg^a^	24.01 (5.75)	26.69 (7.32)	<0.001
Body mass index z-score^a^	−0.16 ± 1.12	0.36 ± 1.13	<0.001
*Cardiovascular measures*			
Systolic blood pressure, mmHg^b^	97 ± 7	111 ± 8	<0.001
Diastolic blood pressure, mmHg^b^	61 ± 5	70 ± 7	<0.001
Mean arterial pressure, mmHg^b^	76 ± 5	87 ± 6	<0.001
Heart rate, bpm^b^	86 ± 11	89 ± 12	<0.001
Pulse wave velocity, m/s^c^	4.38 ± 0.29	4.59 ± 0.32	<0.001
Central retinal artery equivalent, MU^d^	201 ± 15.08	197 ± 14.34	<0.001
Central retinal vein equivalent, MU^d^	236 ± 16.28	236 ± 15.80	0.688
Arterial vein ratio^d^	0.86 ± 0.06	0.84 ± 0.06	<0.001
Cardiorespiratory fitness (stages)^e^	3.287 (1.64)	3.422 (1.47)	0.292
*Food intake frequency per week*			
Fruits	1 (3,5)	1 (3,5)	0.935
Vegetables	1 (3,5)	1 (3,5)	0.154
Meat	3 (5,7)	3 (5,7)	0.287
Milk	1 (5,7)	1 (5,7)	0.249
Cold drinks	1 (5,7)	1 (4,7)	0.506
Sugar tea	1 (3,7)	1 (3,7)	0.588
Cookies/cakes	1 (1,3)	1 (1,3)	0.783
Chips	1 (3,5)	1 (3,5)	0.530
Sweets	1 (3,5)	1 (3,5)	0.457
Fast foods	1 (1,3)	1 (1,3)	0.356
Sugar teaspoons	0 (2,2)	1 (2,2)	0.849

Sample sizes: a = 1043; b = 1040; c = 998; d = 930; e = 646.

P trend values were obtained with the Chi-square test and the Independent T-test. Mean ± SD was given with the p-trend and p-values of the t-test for Equality of Means. For food intake data, percentile ranges: 50th (20th; 80th) obtained from frequency categories of consumption per week. p-values represent comparisons of mean sum ranks between normotensive and incident hypertension groups, using the Mann–Whitney U test for non-parametric independent samples. ANCOVA was used to compare BP with an adjustment applied for body height, sex, and ethnicity, and PWV was additionally adjusted for MAP and HR.

### Factor analysis

**Fig. 1:**
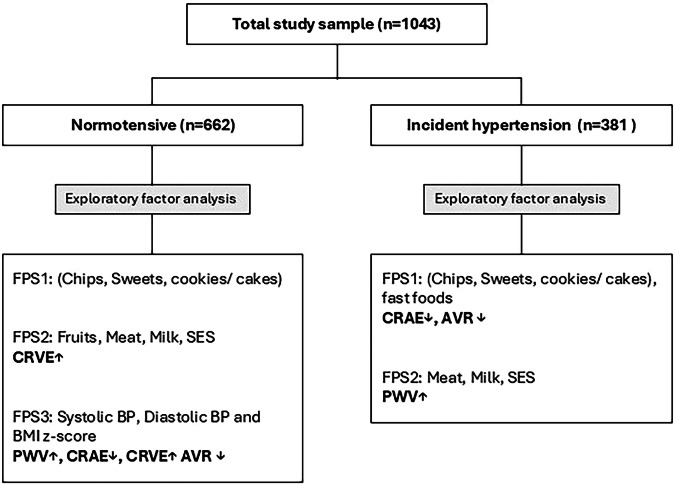
Exploratory factor analysis of food pattern scores and their associations with vascular measures in normotensive and incident hypertension groups. *Fruits only appeared in FPS2 of the normotensive group.

Table 2 contains the factor pattern scores (FPS) from the exploratory factor analysis performed in the normotensive and incident hypertension groups. The analysis identified two similar patterns for both the normotensive group and the incident hypertension groups. The third pattern was identified for only the normotensive group. The first-factor pattern score (FPS1) consisted of chips, sweets, cookies/cakes and fast foods (the latter only in the incident hypertension group); FPS 2 consisted of fruits (only in the normotensive BP group), meat, milk and socioeconomic score; and FPS3 consisted of systolic BP, diastolic BP, and BMI z-score (Fig. 1).

**Table 2:** Factor patterns identified in children with normotensive and incident hypertension.

	Normotensive n = 662			Incident hypertension n = 381	
	FPS1	FPS2	FPS3	FPS1	FPS2
Chips	0.681			0.687	
Sweets	0.671			0.770	
Cookies/cakes	0.602			0.611	
Fast foods				0.507	
Fruits		0.504			
Meat		0.640			0.693
Milk		0.584			0.585
Socioeconomic status score		0.702			0.713
Systolic blood pressure, mmHg			0.725		
Diastolic blood pressure, mmHg			0.663		
Body mass index z-score			0.529		

Extraction Method: Principal Component Analysis. Rotation Method: Oblimin with Kaiser Normalisation.

*FPS* factor pattern score.

### Bivariate and partially adjusted correlations

We performed bivariate correlations of PWV and the retinal markers with each FPS in the normotensive (Supplementary Table 1) and incident hypertension groups (Supplementary Table 2). We performed additional correlation analyses between dietary components, blood pressure, BMI, and vascular measures in normotensive and incident hypertension children (Supplementary Tables 3–6).

In the normotensive group, PWV correlated with FPS3 (r = 0.415; p < 0.001), AVR correlated with FPS2 (r = 0.158, p = 0.028) and FPS3 (r = −0.142; p = 0.048). After adjusting for age, sex, and ethnicity (Table 3), we confirmed the positive correlation between PWV and FPS3 (r = 0.350; p < 0.001), while an inverse correlation between AVR and FPS3 (r = −0.263; p < 0.001) emerged. A positive correlation of CRVE with FPS2 (r = 0.193; p = 0.008) and an inverse correlation of CRAE with FPS3 (r = −0.152; p = 0.0036) were additionally observed.

**Table 3:** Partial correlations of PWV and retinal markers with factor pattern scores for normotensive children.

	PWV (m/s)	CRAE (MU)	CRVE (MU)	AVR
Factor Pattern score 1	r = −0.003	r = −0.118	r = −0.089	r = −0.050
	p = 0.970	p = 0.106	p = 0.225	p = 0.493
Factor Pattern score 2	r = −0.113	r = 0.115	**r** **=** **0.193**	r = −0.064
	p = 0.111	p = 0.116	**p** **=** **0.008**	p = 0.383
Factor Pattern score 3	**r** **=** **0.350**	**r** **=** **−0.152**	r = 0.106	**r** **=** **−0.263**
	**p** **<** **0.001**	**p** **=** **0.036**	p = 0.145	**p** **<** **0.001**

Statistically significant *p* < 0.05 values are in bold.

Adjusted for age, sex, and ethnicity (PWV is additionally adjusted for heart rate and MAP).

*PWV* pulse wave velocity, *CRAE* central retinal artery equivalent, *CRVE* central retinal artery equivalent, *AVR* arterial vein ratio, *r* correlation coefficient.

In the incident hypertension group, there was a positive correlation between PWV and FPS2 (r = −0.261; p = 0.009). CRAE (r = −0.313 p = 0.001) and AVR (r = −0.356; p < 0.001) both correlated inversely with FPS1, while CRVE correlated negatively with FPS2 (r = −0.384; p < 0.001). After adjustments for age, sex, and ethnicity (PWV was additionally adjusted for MAP and HR) (Table 4), we confirmed the previous correlations of AVR (r = −0.343; p < 0.001) and CRAE (r = −0.331; p = 0.001) with FPS1. PWV was positively correlated with FPS2 (r = 0.225, p = 0.029).

**Table 4:** Partial correlations of PWV and retinal markers with factor pattern scores for children with Incident hypertension.

	PWV (m/s)	CRAE (MU)	CRVE (MU)	AVR
Factor pattern score 1	r = 0.123	**r** = **−0.331**	r = −0.013	**r** = **−0.343**
	p = 0.236	**p** = **0.001**	p = 0.879	**p** < **0.001**
Factor pattern score 2	**r** = **0.225**	r = −0.177	r = −0.180	r = −0.118
	**p** = **0.029**	p = 0.087	p = 0.081	p = 0.257

Statistically significant *p* < 0.05 values are in bold.

Adjusted for age, sex, and ethnicity (PWV is additionally adjusted for heart rate and MAP).

*PWV* pulse wave velocity, *CRAE* central retinal artery equivalent, *CRVE* central retinal artery equivalent, *AVR* arterial vein ratio, *r* correlation coefficient.

### Multiple linear regression analysis

**Fig. 2:**
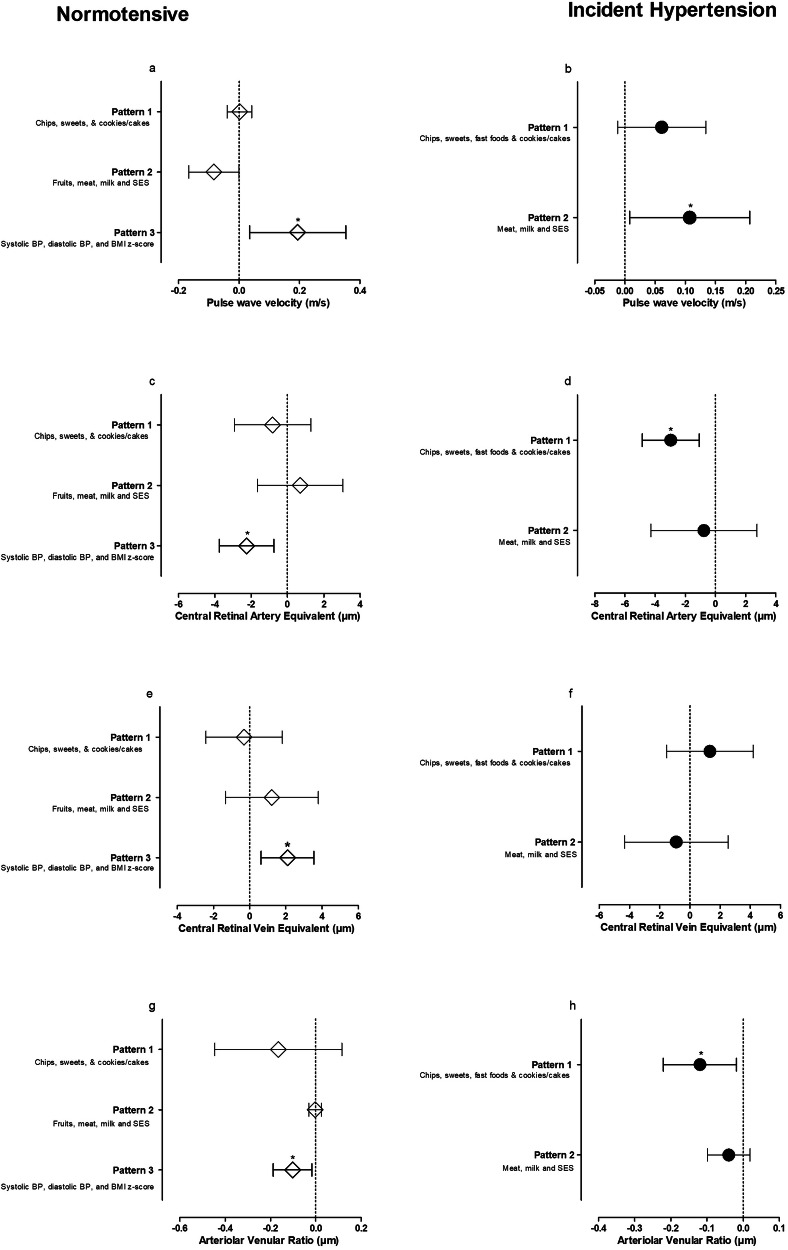
Associations of pulse wave velocity and retinal markers with factor pattern scores in normotensive and incident hypertension groups. Panel **a**–**h** indicate each independent model specific to each blood pressure group and main dependent variable. PWV pulse wave velocity, CRAE central retinal artery equivalent, CRVE central retinal artery equivalent, AVR arterial veinratio, BP blood pressure, SES socio-economic status, BMI z-score Body Mass Index Z-scores.

We performed multiple regression analysis using the backward elimination method to determine the associations between measures of the macro- and microvasculature and the risk factor pattern scores (Fig. 2a–h, Supplementary Tables 7–10). PWV associated with FPS3 (Fig. 2a) in the normotensive group (adj. R^2^ = 0.174, β = 0.372, p < 0.001) and with FPS2 (Fig. 2b) in the incident hypertension group (adj. R^2^ = 0.178, β = 0.197, p = 0.045). CRAE associated with FPS3 (Fig. 2c) in the normotensive group (adj. R^2^ = 0.293, β = −0.224, p = 0.001) and with FPS1 (Fig. 2d) in the incident hypertension group (adj. R^2^ = 0.269, β = −0.329, p < 0.001). CRVE associated with FPS3 (Fig. 2e) in the normotensive group only (adj. R^2^ = 0.359 β = 0.194, p = 0.002) and no association with either FPS1 or FPS2 in the incident hypertension group (Fig. 2f). AVR associated with FPS3 (Fig. 2g) in the normotensive group (adj. R^2^ = 0.158, β = −0.274, p < 0.001) and with FPS1 (Fig. 2h) in the incident hypertension group (adj. R^2^ = 0.22, β = −0.321, p = 0.008).

## Discussion

This study identified cardiovascular risk factor patterns based on BP status and examined associations of macro- and microvasculature measures with the identified risk factor pattern scores in children between 5–9 years old. We observed adverse associations of both micro- and macrovascular health measures with factor patterns consisting of highly processed and fast foods (chips, sweets, and cookies/cakes) as well as meat, milk and SES in children with incident hypertension. Cardiorespiratory fitness was considered for entry into the factor analysis but did not load significantly onto any of the derived patterns, suggesting it did not contribute meaningfully to the risk factor clustering observed in our cohort.

In the incident hypertension group, the two identified food pattern scores reflect distinct but complementary dietary intake patterns. FPS1, comprising chips, sweets, fast foods (incident hypertension group), and cakes/cookies, represents a pattern of highly processed, energy-dense foods that are rich in added sugars, saturated fats, and salt. This pattern was significantly associated with narrower CRAE and lower AVR, markers of adverse microvascular health [24]. FPS2 captures the influence of meat and dairy products that are common among higher SES groups with greater financial ability to access these foods [25]. Notably, FPS2 was significantly associated with increased PWV, suggesting a link to increased arterial stiffness [26]. This reflects the contribution of added salt, sugar and saturated fats in meats and dairy products, alongside the role of SES in shaping access to food quality. It is important to note that fruits were not part of either FPS in the incident hypertension group, limiting any protective dietary influence on vascular outcomes in this subgroup. Together, these findings illustrate that while both FPSs are characterised by processed food consumption, the impact of SES appears more pronounced on large artery stiffness, potentially through enabling access to unfavourable but affordable foods.

Additionally, we found an inverse association of CRAE and AVR with FPS1, suggesting that children consuming high amounts of highly processed foods exhibited narrower retinal arteries. This is consistent with previous studies that link high levels of carbohydrates and sugary soft drinks to narrower retinal arteries in children [27]. Studies suggest that a possible mechanism for these highly processed refined sugar foods leading to narrower retinal arteries is related to metabolic responses to elevated blood sugar levels. A key antioxidant, Glutamate Cysteine Ligase, is mobilised and diverted to metabolise the surplus glucose in the blood [28]. This results in a reduced availability of antioxidants to counteract oxidative stress, while the level of reactive oxygen species increases. Consequently, increased reactive oxygen species lead to vasoconstriction and subsequent arteriolar narrowing and elevated BP [29, 30]. The unhealthy dietary patterns rich in trans fats, sugars and salt may also contribute to systemic inflammation and obesity [31, 32]. This aligns with findings from a previous study that also found that narrower arterioles were associated with obesity and elevated BP, indicative of early CVD development [33]. The narrowing of retinal arterioles observed in our sample of children is particularly concerning, as it serves as an early indicator of vascular dysfunction strongly related to modifiable risk factors [34].

In the normotensive group, we observed positive associations of both PWV and AVR with FPS3, which comprised systolic BP, diastolic BP and BMI. In addition, both CRAE and AVR associated inversely with FPS3. These associations seem physiologically feasible in the normotensive context whereby the main component for PWV is BP [13, 24]. Similarly for the microvascular measures it is known that higher BP contribute to arteriolar narrowing of the retina. Both the average BMI z-scores and the BIA-derived fat mass index data (Supplementary Table 11) indicate that widespread excess adiposity was not present in our sample. Nevertheless, the positive association with PWV suggests that variations in BMI, even within the slightly elevated levels, can still influence arterial structure and function. This indicates that children with BP levels below clinical concern display the physiological dynamics of BP and body composition on vascular health preservation.

Our findings have shown that the combined effect of unhealthy diet and SES was observed only in children with incident hypertension, not in the normotensive group. This suggests that these factors are particularly relevant in the development of hypertension, demonstrating the need for healthier dietary choices in this group. In normotensive children, traditional risk factors such as genetic predisposition, birth weight, BP, and adiposity primarily drive macro- and microvascular alterations [35]. However, among children in our study with incident hypertension, modifiable factors, particularly unhealthy dietary intake, appear to play a more dominant role in early vascular compromise. The observed associations of PWV, CRAE, and AVR with unhealthy dietary intake, and at times SES, suggest that poor nutrition, compounded by socioeconomic disparities, may accelerate vascular dysfunction in children with incident hypertension as early as five years of age. Our observations demonstrate the need for further investigation into the potential protective effects of wholesome dietary patterns, such as fruits, on vascular health in this population and context.

This study has strengths and limitations to report. This paper reports on a cross-sectional analysis using only baseline data from the ExAMIN Youth SA study, a prospective cohort study [36]. While this allowed us to explore associations between dietary intake patterns, vascular measures, and BP status, the cross-sectional nature of the analysis limits our ability to establish causality. Additionally, we recruited children in the North-West province of South Africa from public schools that were in urban areas, which may not reflect the sociodemographic profile of the entire country. Despite these limitations, this study made use of a Static Retinal Vessel Analyser to non-invasively observe the health status of the retinal microvasculature in children with minimal discomfort. To the best of our knowledge, this is the first study to assess both macro- and microvascular health measures with composite risk factor patterns to determine the modifiable contributors to incident hypertension risk in children under 9 years of age.

Our findings emphasise the importance of screening children from an early age for blood pressure, as those with incident hypertension appeared more susceptible to the combined influence of poor diet. This enables targeted lifestyle interventions, particularly dietary modifications that can alleviate risk. We recommend the promotion of healthier food intake patterns, alongside nutrition education, and improving access to nutritious foods. Early intervention strategies that target both dietary habits are vital to breaking the cycle of cardiovascular disease onset in children that may track into adulthood.

## Conclusion

Our study established a significant association between frequent consumption of diets high in fast foods, meat, and milk, alongside household socioeconomic factors, and compromise in micro- and macrovascular health in children with incident hypertension.

## Summary

### What is known about the topic

1. Early exposure to cardiovascular risk factors such as obesity, elevated blood pressure and unhealthy dietary patterns is associated with adverse vascular changes in children.
2. Retinal vessel diameters and pulse wave velocity are validated early markers of vascular dysfunction and predictors of future cardiovascular disease.
3. Socioeconomic status is a key determinant of dietary behaviour, which in turn affects cardiovascular risk trajectories from a young age.

### What this study adds

1. Distinct patterns of dietary intake and socioeconomic factors are differentially associated with macro- and microvascular alterations in children, depending on their blood pressure status.
2. Among children with incident hypertension, unhealthy dietary patterns showed stronger associations with vascular dysfunction, as reflected by narrower arteriolar diameters and increased arterial stiffness.
3. The findings highlight the importance of early screening and targeted dietary interventions to mitigate vascular compromise and reduce long-term cardiovascular risk.

## Supplementary information

Associations of Cardiovascular and Retinal Markers with Risk Factor Patterns in Children with and without Incident Hypertension

## Data Availability

The data used in this study are not publicly however, anonymised summary data relevant to the analyses presented in this manuscript can be made available upon reasonable request to the corresponding author, subject to institutional and ethical approvals.
