# Supplementary material for: Risk factor patterns and vascular health in children with incident hypertension: The ExAMIN Youth SA study
Source: J Hum Hypertens. 2025 Oct 2;39(12):859–66. doi: 10.1038/s41371-025-01074-w (PMC12685736; doi:10.1038/s41371-025-01074-w)
Supplement: Supplementary file 1 — Associations of Cardiovascular and Retinal Markers with Risk Factor Patterns in Children with and without Incident Hypertension [file 41371_2025_1074_MOESM1_ESM.docx]

**Supplementary Table 1:** Pearson correlations of PWV and retinal markers with factor pattern scores for normotensive children

|  | **PWV (m/s)** | **CRAE (MU)** | **CRVE (MU)** | **AVR** |
| --- | --- | --- | --- | --- |
| Factor Pattern score 1 | r = -0.081 | r = -0.114 | r = -0.035 | r = -0.091 |
|  | p = 0.248 | p = 0.114 | p = 0.624 | p = 0.207 |
| Factor Pattern score 2 | r = -0.029 | r = 0.082 | r = -0.084 | **r = 0.158** |
|  | p = 0.677 | p = 0.255 | p = 0.242 | **p = 0.028** |
| Factor Pattern score 3 | **r = 0.415** | r = -0.110 | r = -0.029 | **r = -0.142** |
|  | **p < 0.001** | p = 0.128 | p = 0.686 | **p = 0.048** |

Abbreviations: PWV - pulse wave velocity, CRAE - central retinal artery equivalent, CRVE - central retinal artery equivalent, AVR - arterial vein ratio, r - correlation coefficient.

**Supplementary Table 2:** Pearson correlations of PWV and retinal markers with factor pattern scores for children with incident hypertension

|  | **PWV (m/s)** | **CRAE (MU)** | **CRVE (MU)** | **AVR** |
| --- | --- | --- | --- | --- |
| Factor Pattern score 1 | r = 0.025 | **r = -0.313** | r = 0.051 | **r = -0.356** |
|  | p = 0.801 | **p = 0.001** | p = 0.612 | **p < 0.001** |
| Factor Pattern score 2 | **r = 0.261** | r = -0.134 | **r = -0.384** | r = 0.159 |
|  | **p = 0.009** | p = 0.181 | **p = < 0.001** | p = 0.113 |
|  |  |  |  |  |

Abbreviations: PWV - pulse wave velocity, CRAE - central retinal artery equivalent, CRVE - central retinal artery equivalent, AVR - arterial vein ratio, r - correlation coefficient.

**Supplementary Table 3:** Pearson correlations of PWV and retinal markers with individual factor components for normotensive children

|  | **PWV (m/s)** | **CRAE (MU)** | **CRVE (MU)** | **AVR** |
| --- | --- | --- | --- | --- |
| Chips | r = -0.006 | r = -0.084 | r = 0.068 | **r = -0.150** |
|  | p = 0.883 | p = 0.05 | p = 0.122 | **p < 0.001** |
| Sweets | r = 0. 011 | r = -0.027 | r = -0.029 | r = -0.005 |
|  | p = 0.801 | p = 0.535 | p = 0.513 | p = 0.914 |
| Cookies/cakes | r = 0.047 | r = -0.038 | r = 0.033 | r = -0.066 |
|  | p = 0.268 | p = 0.390 | p = 0.455 | p = 0.136 |
| Fruits | r = -0.046 | r = 0.047 | r = 0.008 | r = 0.045 |
|  | p = 0.277 | p = 0.287 | p = 0.852 | p = 0.307 |
| Meat | r = 0.043 | r = 0.058 | **r = -0.128** | **r = 0.173** |
|  | p = 0.318 | p = 0.189 | **p = 0.004** | **p < 0.001** |
| Vegetables | **r = -0.090** | **r = 0.095** | r = 0.009 | **r = 0.091** |
|  | **p = 0.032** | **p = 0.029** | p = 0.840 | **p = 0.037** |
| Milk | r = 0.053 | r = 0.015 | **r = -0.092** | **r = 0.104** |
|  | p = 0.211 | p = 0.737 | **p = 0.035** | **p = 0.018** |
| Systolic BP, mmHg | **r = 0.392** | **r = -0.099** | r = -0.021 | **r = -0.084** |
|  | **p < 0.001** | **p = 0.016** | p = 0.617 | **p = 0.042** |
| Diastolic BP, mmHg | **r = 0.240** | **r = -0.124** | r = -0.033 | **r = -0.090** |
|  | **p < 0.001** | **p = 0.003** | p = 0.428 | **p = 0.029** |
| BMI z-score | **r = 0.168** | r = -0.071 | **r = -0.085** | r = 0.003 |
|  | **p < 0.001** | p = 0.084 | **p = 0.039** | p = 0.933 |

Abbreviations: PWV - pulse wave velocity, CRAE - central retinal artery equivalent, CRVE - central retinal artery equivalent, AVR - arterial vein ratio, BP - blood pressure, BMI - body mass index, r - correlation coefficient.

**Supplementary Table 4:** Partial correlations of PWV and retinal markers with individual factor components for normotensive children

|  | **PWV (m/s)** | **CRAE (MU)** | **CRVE (MU)** | **AVR** |
| --- | --- | --- | --- | --- |
| Chips | r = 0.053 | r = -0.076 | r = -0.015 | r = -0.069 |
|  | p = 0.217 | p = 0.085 | p = 0.738 | p = 0.117 |
| Sweets | r = 0.016 | r = -0.040 | r = -0.044 | r = -0.004 |
|  | p = 0.703 | p = 0.367 | p = 0.315 | p = 0.921 |
| Cookies/cakes | r = 0.059 | r = -0.024 | r = 0.012 | r = -0.033 |
|  | p = 0.169 | p = 0.588 | p = 0.794 | p = 0.455 |
| Fruits | r = -0.042 | r = 0.028 | r = 0.001 | r = 0.035 |
|  | p = 0.321 | p = 0.523 | p = 0.981 | p = 0.426 |
| Meat | r = 0.017 | r = -0.037 | r = -0.041 | r = 0.073 |
|  | p = 0.685 | p = 0.397 | p = 0.359 | p = 0.099 |
| Vegetables | r = -0.063 | r = 0.094 | r = 0.020 | r = 0.083 |
|  | p = 0.141 | p = 0.032 | p = 0.651 | p = 0.057 |
| Milk | r = -0.005 | r = -0.017 | r = 0.020 | r = -0.033 |
|  | p = 0.912 | p = 0.697 | p = 0.645 | p = 0.455 |
| Systolic BP, mmHg | **r = 0.137** | **r = -0.99** | r = 0.024 | **r = -0.131** |
|  | **p < 0.001** | **p = 0.016** | p = 0.566 | **p = 0.001** |
| Diastolic BP, mmHg | **r = -0.142** | **r = -0.128** | r = -0.061 | r = -0.073 |
|  | **p < 0.001** | **p = 0.002** | p = 0.139 | p = 0.077 |
| BMI z-score | **r = 0.092** | **r = -0.093** | r = -0.050 | r = -0.055 |
|  | **p = 0.022** | **p = 0.025** | p = 0.228 | p = 0.584 |

Adjusted for age, sex, and ethnicity (PWV is additionally adjusted for heart rate and MAP)

Abbreviations: PWV - pulse wave velocity, CRAE - central retinal artery equivalent, CRVE - central retinal artery equivalent, AVR - arterial vein ratio, BP - blood pressure, BMI - body mass index, r - correlation coefficient.

**Supplementary Table 5:** Pearson correlations of PWV and retinal markers with individual factor components for children with incident hypertension

|  | **PWV (m/s)** | **CRAE (MU)** | **CRVE (MU)** | **AVR** |
| --- | --- | --- | --- | --- |
| Chips | r = 0.029 | r = -0.087 | r = 0.088 | **r = -0.172** |
|  | p = 0.605 | p = 0.130 | p = 0.126 | **p = 0.003** |
| Sweets | r = 0.019 | r = -0.057 | r = -0.055 | r = -0.014 |
|  | p = 0.738 | p = 0.318 | p = 0.342 | p = 0.808 |
| Cookies/cakes | r = -0.002 | r = -0.068 | r = 0.073 | **r = -0.126** |
|  | p = 0.975 | p = 0.245 | p = 0.205 | **p = 0.029** |
| Fruits | r = -0.050 | r = 0.030 | r = -0.029 | r = 0.046 |
|  | p = 0.365 | p = 0.595 | p = 0.612 | p = 0.420 |
| Meat | **r = 0.155** | r = -0.056 | **r = -0.157** | r = 0.090 |
|  | **p = 0.005** | p = 0.329 | **p = 0.006** | p = 0.117 |
| Vegetables | r = -0.085 | r = -0.009 | r = -0.062 | r = 0.046 |
|  | p = 0.126 | p = 0.881 | p = 0.280 | p = 0.421 |
| Milk | r = 0.020 | r = -0.105 | **r = -0.205** | r = 0.085 |
|  | p = 0.724 | p = 0.067 | **p < 0.001** | p = 0.140 |
| Systolic BP, mmHg | **r = 0.475** | r = -0.044 | r = -0.052 | r = 0.002 |
|  | **p < 0.001** | p = 0.415 | p = 0.334 | p = 0.966 |
| Diastolic BP, mmHg | **r = 0.119** | **r = -0.170** | **r = 0.110** | **r = -0.252** |
|  | **p = 0.023** | **p = 0.002** | **p = 0.042** | **p < 0.001** |
| BMI z-score | **r = 0.258** | r = -0.054 | r = -0.041 | r = -0.011 |
|  | **p < 0.001** | p = 0.317 | p = 0.446 | p = 0.837 |

Abbreviations: PWV - pulse wave velocity, CRAE - central retinal artery equivalent, CRVE - central retinal artery equivalent, AVR - arterial vein ratio, BP - blood pressure, BMI - body mass index, r - correlation coefficient.

**Supplementary Table 6:** Partial correlations of PWV and retinal markers with individual factor components for children with incident hypertension

|  | **PWV (m/s)** | **CRAE (MU)** | **CRVE (MU)** | **AVR** |
| --- | --- | --- | --- | --- |
| Chips | r = 0.072 | r = -0.093 | r = -0.008 | **r = -0.116** |
|  | p = 0.199 | p = 0.107 | p = 0.895 | **p = 0.045** |
| Sweets | r = 0.001 | r = -0.062 | r = -0.083 | r = 0.000 |
|  | p = 0.987 | p = 0.285 | p = 0.151 | p = 0.994 |
| Cookies/cakes | r = 0.025 | r = -0.078 | r = 0.001 | r = -0.078 |
|  | p = 0.664 | p = 0.182 | p = 0.981 | p = 0.181 |
| Fruits | r = -0.001 | r = 0.018 | r = -0.042 | r = 0.047 |
|  | p = 0.980 | p = 0.751 | p = 0.462 | p = 0.412 |
| Meat | r = 0.940 | r = -0.061 | r = -0.008 | r = -0.048 |
|  | p = 0.092 | p = 0.294 | p = 0.892 | p = 0.407 |
| Vegetables | r = -0.052 | r = -0.018 | r = -0.052 | r = 0.028 |
|  | p = 0.353 | p = 0.760 | p = 0.368 | p = 0.631 |
| Milk | r = -0.003 | **r = -0.129** | r = -0.077 | r = -0.058 |
|  | p = 0.954 | **p = 0.025** | p = 0.182 | p = 0.312 |
| Systolic BP, mmHg | **r = 0.271** | r = -0.020 | r = 0.032 | r = -0.051 |
|  | **p < 0.001** | p = 0.717 | p = 0.560 | p = 0.354 |
| Diastolic BP, mmHg | **r = -0.262** | **r = -0.175** | r = 0.019 | **r = -0.189** |
|  | **p < 0.001** | **p = 0.001** | p = 0.733 | **p < 0.001** |
| BMI z-score | **r = 0.131** | r = -0.046 | r = -0.073 | r = -0.105 |
|  | **p = 0.012** | p = 0.402 | p = 0.179 | p = 0.053 |

Adjusted for age, sex, ethnicity (PWV is additionally adjusted for heart rate and MAP)

Abbreviations: PWV - pulse wave velocity, CRAE - central retinal artery equivalent, CRVE - central retinal artery equivalent, AVR - arterial vein ratio, BP - blood pressure, BMI - body mass index, r - correlation coefficient.

**Supplementary Table 7**: Multiple regression analysis of pulse wave velocity and risk factor pattern scores

| **Blood pressure category** | | | | | | |
| --- | --- | --- | --- | --- | --- | --- |
|  |  | **Normotensive (n =662)** | |  | **Incident Hypertension (n=381)** | |
|  | **Adj. R^2^** | **Std β (±95 Cl)** | **p** | **Adj. R^2^** | **Std β (±95 Cl)** | **p** |
|  |  |  |  |  |  |  |
| Factor Pattern score 1 | 0.153 | 0.003 (-0.040; 0.041) | 0.970 | 0.160 | 0.115 (-0.022;0.090) | 0.236 |
| Ethnicity |  | 0.098 (-0.020; -0.136) | 0.146 |  | 0.137 (-0.037;0.216) | 0.162 |
| Sex |  | 0.081 (-0.027;0.123) | 0.211 |  | -0.018 (-0.132;0.108) | 0.847 |
| Age, years |  | 0.154 (0.006;0.088) | 0.024 |  | 0.181 (-0.04;0.141) | 0.065 |
| HR, bpm |  | 0.070 (-0.002; 0.005) | 0.294 |  | 0.058 (-0.125;0.238) | 0.540 |
| MAP, mmHg |  | 0.304 (0.009;0.025) | < 0.001 |  | 0.332 (0.027;0.092) | 0.003 |
| BMI-z |  | 0.087 (-0.012; 0.058) | 0.199 |  | 0.140 (-0.017; 0.97) | 0.164 |
|  |  |  |  |  |  |  |
| Factor Pattern score 2 | 0.168 | -0.163 (-0.090;0.003) | 0.060 | 0.178 | **0.197 (0.000; 0.125)** | **0.049** |
| Ethnicity |  | 0.185 (0.016;0.202) | 0.022 |  | 0.008 (-0.148;0.158) | 0.947 |
| Sex |  | 0.85 (-0.024;0.125) | 0.186 |  | -0.036 (-0.143;0.096) | 0.697 |
| Age, years |  | 0.146 (0.004;0.086) | 0.030 |  | 0.190 (0.000;0.144) | 0.051 |
| MAP, mmHg |  | 0.302 (0.010;0.025) | < 0.001 |  | 0.305 (0.006;0.029) | 0.003 |
| BMI-z |  | 0.076 (-0.015; 0.054) | 0.256 |  | 0.118 (-0.021;0.089) | 0.227 |
| HR, bpm |  | 0.038 (-0.003, 0.005) | 0.575 |  |  |  |
|  |  |  |  |  |  |  |
| Factor Pattern score 3* | 0.174 | **0.372 (0.066;0.144)** | **< 0.001** | N/A | N/A | N/A |
| Ethnicity |  | 0.020 (-0.067;0.090) | 0.769 |  | N/A | N/A |
| Sex |  | 0.076 (-0.029;0.119) | 0.234 |  | N/A | N/A |
| Age, years |  | 0.133 (0.00;0.082) | 0.050 |  | N/A | N/A |
| HR, bpm |  | 0.053 (-0.002,0.005) | 0.418 |  | N/A | N/A |
|  |  |  |  |  |  |  |

Abbreviations: Adj. - adjusted, Std b - standardised beta, HR - heart rate, bpm - beats per minute, MAP - mean arterial pressure.

* Indicates significant risk factor pattern scores.

**Supplementary Table 8**: Multiple regression analysis of CRAE and risk factor pattern scores

| **Blood pressure category** | | | | | | |
| --- | --- | --- | --- | --- | --- | --- |
|  |  | **Normotensive (n =662)** | |  | **Incident Hypertension (n=381)** | |
|  | **Adj. R^2^** | **Std β (±95 Cl)** | **p** | **Adj. R^2^** | **Std β (±95 Cl)** | **p** |
|  |  |  |  |  |  |  |
| Factor Pattern score 1* | 0268 | -0.076 (-3.203, 0.822) | 0.245 | 0.269 | **-0.329 (-6.605;-1.951)** | **< 0.001** |
| Ethnicity |  | 0.227 (2.821;10.956) | 0.001 |  | 0.144 (-1.618;9943) | 0.156 |
| Sex |  | -0.083 (-6.335, 1.275) | 0.191 |  | -0.055 (-6.68, 3.423) | 0.533 |
| Age, years |  | -0.025 (-2.432, 1.631) | 0.698 |  | -0.055 (-3.973; 2.151) | 0.556 |
| MAP, mmHg |  | -0.120 (-1.038,-0.338) | 0.316 |  | -0.253 (-1.250, -0.035) | 0.039 |
| BMI-z |  | -0.034 (-2.195;1.277) | 0.602 |  | -0.089 -3.493, 1.245) | 0.349 |
|  |  |  |  |  |  |  |
| Factor Pattern score 2 | 0.261 | 0.069 (--1.286, 3.300) | 0.387 | 0.166 | -0.079 (-4.576, 2.356) | 0.526 |
| Ethnicity |  | 0.202 (1.215, 11.057) | 0.015 |  | 0.241 (-0.178, 14.11) | 0.056 |
| Sex |  | -0.086 (-6.436, 1.199) | 0.178 |  | - 0.038 (-6.436, 4.265) | 0.688 |
| Age, years |  | -0.024 (-2.415, 1.663) | 0.716 |  | -0.061 (-4.289, 2.270) | 0.542 |
| MAP, mmHg |  | -1.02 (-0.981, 0.386) | 0.391 |  | -0.260 (-1.309, -0.008) | 0.047 |
| BMI-z |  | -0.038 (-2.246, 1.221) | 0.560 |  | -0.053 (-3.204, 1.852) | 0.597 |
|  |  |  |  |  |  |  |
| Factor Pattern score 3* | 0.293 | **-0.224 (-5.192, -1.319)** | **0.001** | N/A | N/A | N/A |
| Ethnicity |  | 0.303 (5.150, 13.215) | < 0.001 |  | N/A | N/A |
| Sex |  | -0.080 (-6.112, 1.271) | 0.197 |  | N/A | N/A |
| Age, years |  | 0.001 (-2.004, 2.021) | 0.993 |  | N/A | N/A |

Abbreviations: Adj. - adjusted, Std b - standardised beta, MAP - mean arterial pressure. * Indicates significant risk factor pattern scores.

**Supplementary Table 9:** Multiple regression analysis of CRVE and risk factor pattern scores

| **Blood pressure category** | | | | | | |
| --- | --- | --- | --- | --- | --- | --- |
|  |  | **Normotensive (n =662)** | |  | **Incident Hypertension (n=381)** | |
|  | **Adj. R^2^** | **Std β (±95 Cl)** | **p** | **Adj. R^2^** | **Std β (±95 Cl)** | **p** |
|  |  |  |  |  |  |  |
| Factor Pattern score 1 | 0.307 | -0.026 (-2.539, 1.656) | 0.678 | 0.286 | 0.134 (-0.751, 4.600) | 0.157 |
| Ethnicity |  | -0.319 (-14.534, -6.355) | < 0.001 |  | -0.405 (-18.552, -7.171) | < 0.001 |
| Sex |  | -0.090 (6.887, 1.020) | 0.145 |  | -0.051 (-6.978, 3.782) | 0.557 |
| Age, years |  | 0.028 (-1.640, 2.585) | 0.659 |  | 0.017 (-3.032, 3.644) | 0.856 |
| BMI-z |  | -0.008 (-1.932, 1.703) | 0.901 |  | 0.100 (-1.181, 3.960) | 0.286 |
|  |  |  |  |  |  |  |
| Factor Pattern score 2* | 0.314 | 0.113 (-0.594, 4.161) | 0.141 | 0.290 | -0.084 (-4.668, 2.042) | 0.439 |
| Ethnicity |  | -0.378 (-17.240, -7.503) | < 0.001 |  | -0.376 (-18.899, -5.005) | < 0.001 |
| Sex |  | -0.095 (-7.035, 0.840) | 0.122 |  | -0.052 (-7.041, 3.770) | 0.549 |
| Age, years |  | 0.033 (-1.541, 2.669) | 0.598 |  | 0.009 (-3.090, 3.432) | 0.917 |
| BMI-z |  | -0.003 (-1845, 1.762) | 0.964 |  | 0.089 (-1.323, 3.808) | 0.339 |
|  |  |  |  | N/A |  |  |
| Factor Pattern score 3 | 0.359 | **0.194 (1.127, 4.957)** | **0.002** |  | N/A | N/A |
| Ethnicity |  | -0.371 (-16.134, -8.142) | < 0.001 |  | N/A | N/A |
| Sex |  | -0.078 (-6.382, 1.289) | 0.192 |  | N/A | N/A |
|  |  |  |  |  |  |  |

Abbreviations: Adj. - adjusted, Std b - standardised beta. * Indicates significant risk factor pattern scores.

**Supplementary Table 10**: Multiple regression analysis of AVR and risk factor patterns

| **Blood pressure category** | | | | | | |
| --- | --- | --- | --- | --- | --- | --- |
|  |  | **Normotensive (n =662)** | |  | **Incident Hypertension (n=381)** | |
|  | **Adj. R^2^** | **Std β (±95 Cl)** | **p** | **Adj. R^2^** | **Std β (±95 Cl)** | **p** |
|  |  |  |  |  |  |  |
| Factor Pattern score 1 | 0.109 | -0.49 (-0.013, 0.006) | 0.491 | 0.220 | **-0.321 (-0.029; -0.008)** | **< 0.001** |
| Ethnicity |  | 0.348 (0.026, 0.063) | < 0.001 |  | 0.309 (0.015, 0.065) | 0.002 |
| Sex |  | -0.005 (-0.18;0.017) | 0.947 |  | -0.021 (-0.026; 0.020) | 0.813 |
| Age, years |  | -0.03 (-0.011, 0.007) | 0.638 |  | -0.039 (-0.017, 0.011) | 0.680 |
| MAP, mmHg |  | -0.037 (-0.004, 0.003) | 0.775 |  | -0.235 (-0.005, 0.000) | 0.060 |
| BMI-z |  | -0.024 (-0.009, 0.007) | 0.734 |  | -0.109 (-0.017;0.005) | 0.264 |
|  |  |  |  |  |  |  |
| Factor Pattern score 2 | 0.107 | -0.008 (-0.011, 0.010) | 0.923 | 0.140 | -0.106 (-0.021, 0.008) | 0.373 |
| Ethnicity |  | 0.363 (0.025, 0.068) | < 0.001 |  | 0.421 (0.023, 0.085) | < 0.001 |
| Sex |  | -0.005 (-0.018, 0.017) | 0.942 |  | -0.04 (-0.025, 0.024) | 0.968 |
| Age, years |  | -0.035 (-0.012, 0.007) | 0.619 |  | -0.054 (-0.019, 0.003) | 0.581 |
| MAP, mmHg |  | -0.027 (-0.004, 0.003) | 0.833 |  | -0.238 (-0.66;0.000) | 0.069 |
| BMI-z |  | -0.031 (-0.010, 0.006) | 0.662 |  | -0.78 (-0.016, 0.007) | 0.442 |
|  |  |  |  | N/A |  |  |
| Factor Pattern score 3* | 0.158 | **-0.274 (-0.026, -0.008)** | **< 0.001** |  | N/A | N/A |
| Ethnicity |  | 0.422 (0.036, 0.072) | < 0.001 |  | N/A | N/A |
| Sex |  | -0..014 (-0.019, 0.015) | 0.836 |  | N/A | N/A |
| Age, years |  | 0.012 (-0.008, 0.010) | 0.861 |  | N/A | N/A |

Abbreviations: Adj. - adjusted, Std b - standardised beta, MAP - mean arterial pressure. * Indicates significant risk factor pattern scores.

**Supplementary Table 11:** Distribution of adiposity in the sample

| Outcome | Frequency | Percentage (%) |
| --- | --- | --- |
| BMI percentiles^1^  Severely underweight  Underweight  Normal  Overweight  Obese  Severely Obese | 10  29  806  157  45  01 | 0.9  2.7  75.5  14.7  4.2  0.1 |
| Fat Mass & Fat Free Mass Indices^2^  Normal adiposity  High adiposity | 994  73 | 93.2  6.8 |

^1^: 19 missing values. BMI percentiles as per the WHO classification.

^2^: Comparison against Body-composition reference data (Wells *et al.*, 2012).

Fat mass index (x̄= 4.51, SD= 1.99), Fat Free Mass Index (x̄= 11.81, SD= 0.94).
